# Interruption of the right middle and lower lobe pulmonary arteries combined with arterial aneurysm formation at the origin of the right subclavian artery branch: a case report

**DOI:** 10.3389/fcvm.2026.1821385

**Published:** 2026-05-18

**Authors:** Chengshi Hou, Guocheng Zhao

**Affiliations:** The Fourth People's Hospital of Chengdu, Chengdu, China

**Keywords:** abnormal development, case report, computed tomography angiography, proximal interruption of pulmonary artery, pulmonary hypertension

## Abstract

This paper reports a rare adult case of a 64-year-old female with interrupted pulmonary artery in the right middle and lower lobes, complicated by a branch aneurysm at the origin of the right subclavian artery. The patient was admitted for “depressed mood.” And she experienced intermittent chest tightness and mild shortness of breath after physical activity. Elevated D-dimer levels were detected, and CT pulmonary angiography confirmed congenital interruption of the right middle and lower lobe pulmonary arteries, accompanied by extensive collateral circulation formation and pulmonary hypertension. This condition originates from abnormal development of the sixth aortic arch during embryogenesis and may clinically mimic chronic pulmonary embolism. Imaging evaluation is central to diagnosis, and treatment requires individualization: asymptomatic patients should undergo conservative follow-up, while those developing hemoptysis or progressive pulmonary hypertension may require interventional embolization or surgical intervention. This case underscores the need for vigilance regarding such rare vascular anomalies, where early recognition and long-term follow-up are crucial for improving patient prognosis.

## Introduction

Proximal interruption of the pulmonary artery (PIPA) is a rare congenital anomaly, predominantly observed in children, with Isolated unilateral interruption of pulmonary artery occurring at a rate of approximately 1 in 200,000 (1, 2). Right middle and lower lobe pulmonary artery interruption represents a specific subtype of PIPA, characterized by congenital interruption of the distal branches (middle and lower lobe arteries) of the right pulmonary artery trunk, while the proximal trunk and upper lobe branches remain intact. This condition typically arises from failure of the sixth aortic arch to connect with the pulmonary vascular bed during embryogenesis (3, 4). It is often associated with congenital heart disease and frequently involves collateral circulation formation. Clinically, it is easily misdiagnosed as chronic pulmonary embolism or bronchiectasis. Common symptoms include recurrent pulmonary infections, exertional dyspnea, reduced exercise tolerance, chest pain, and hemoptysis. Currently, no definitive surgical treatment exists for pulmonary artery interruption. Some scholars advocate conservative observation for asymptomatic patients; however, if recurrent hemoptysis or pulmonary hypertension occurs, interventional embolization or surgical resection of the affected lobe should be considered (5). Terminology such as “absence, atresia, and interruption” is used; ‘absence’ and “atresia” are not entirely accurate since other vessels supply blood to the lung tissue (4). This paper discusses the anatomical characteristics, radiological diagnosis, and treatment options for pulmonary artery interruption in an adult patient with right middle and lower lobe involvement, supplemented by relevant literature.

## Case description

Female, 64 years old, admitted for “low mood, depressive episode.” The patient experienced intermittent chest tightness and mild shortness of breath after physical activity. History of hypertension; denies heart disease, diabetes, or cerebrovascular disease; no smoking history.

On admission, patient had elevated blood pressure (159/92 mmHg), regular heart rate of 88 beats per minute, no dyspnea at rest, respiratory rate 20 breaths per minute.

Chest and cardiac examinations were unremarkable; no cyanosis was observed. Jugular veins were normal without distension, and the jugular venous pulse was absent.

Admission laboratory tests included: complete blood count, normal liver and kidney function, blood glucose, and lipid levels within normal ranges. The cardiac infarction triad test showed no abnormalities. Due to the patient's intermittent chest tightness and mild shortness of breath after physical activity, a D-dimer test was performed. Elevated D-dimer at 2.94 μg/mL (reference range <1 μg/mL) prompted CT pulmonary angiography (CTPA).

ECG showed sinus rhythm with frequent atrial premature beats and flattened T waves.

**Figure 1:**
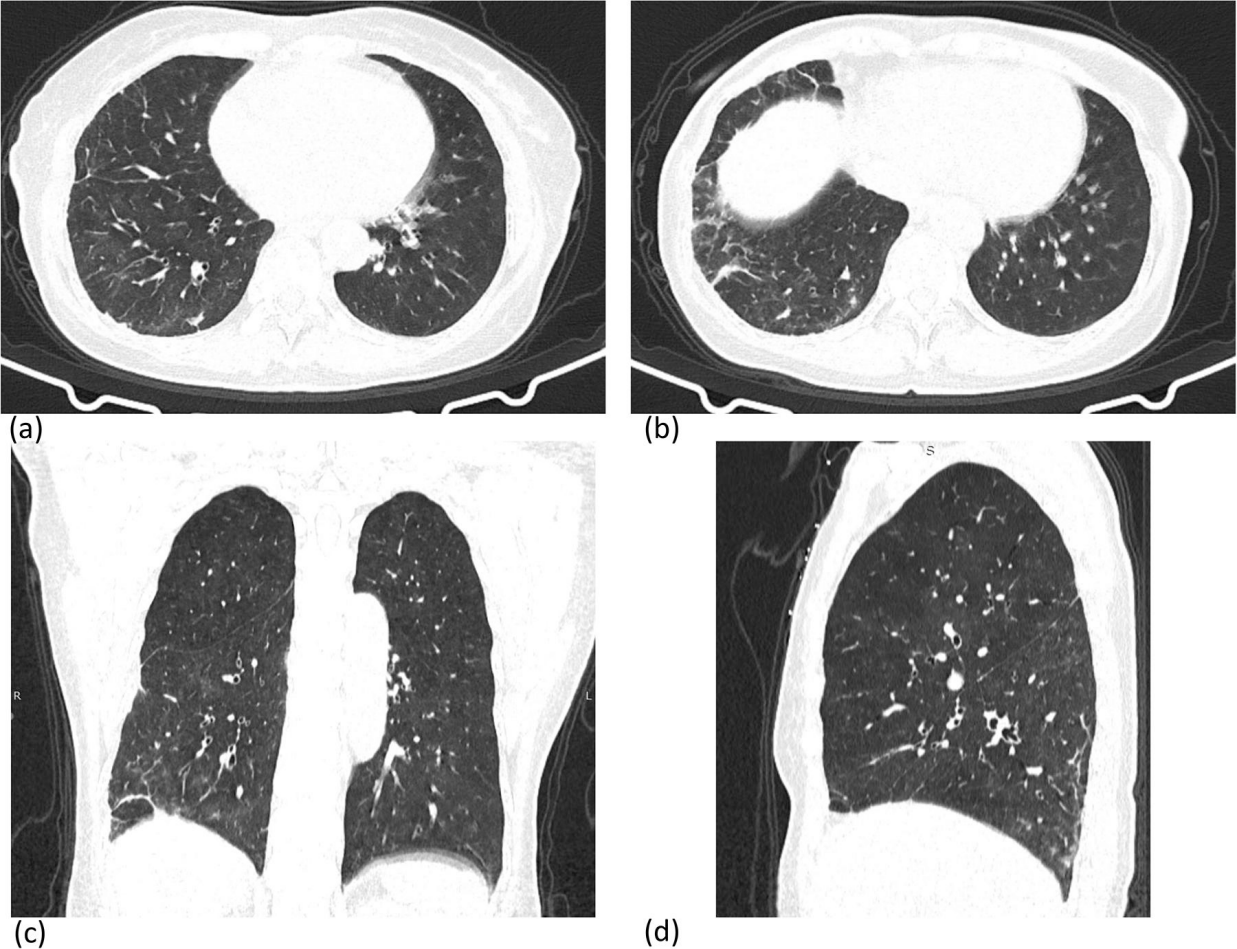
Chest CT revealed linear opacities in the right middle and lower lobes with interlobular septal thickening, suggestive of pulmonary interstitial changes **(a,b)** axial; **(c,d)** coronal reconstruction.

Chest CT revealed linear opacities in the right middle and lower lobes with interlobular septal thickening, suggestive of pulmonary interstitial changes (Figure 1).

Cardiac ultrasound revealed: enlarged left atrium, dilated pulmonary artery trunk and branches, mild mitral and tricuspid regurgitation. Abnormal blood flow signals within the pulmonary artery lumen suggested possible patent ductus arteriosus.

**Figure 2:**
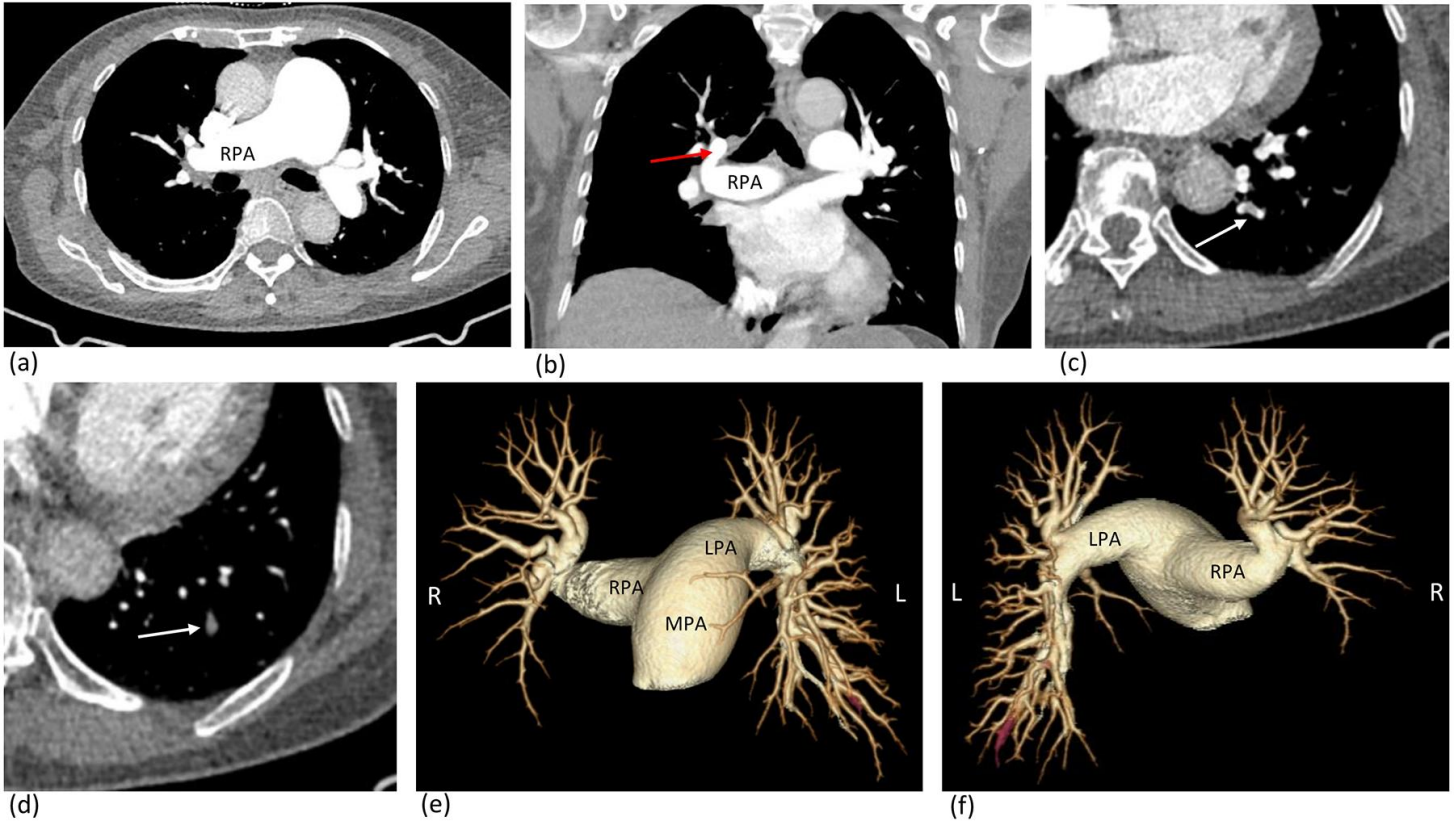
Axial images from contrast-enhanced CT scans **(a,b,c,d)** and 3D reconstruction **(e,f)** shows interruption of the pulmonary artery in the right middle and lower lobes, with dilation of the pulmonary artery trunk and left pulmonary artery and pulmonary embolism on distal branch of the left lower lobe pulmonary arteries. Pulmonary embolism indicated by white arrow **(c,d)** and red highlight **(e,f)**. Pulmonary artery in the right upper lobes indicated by red arrow. LPA left pulmonary artery; RPA right pulmonary artery; MPA main pulmonary artery.

**Figure 3:**
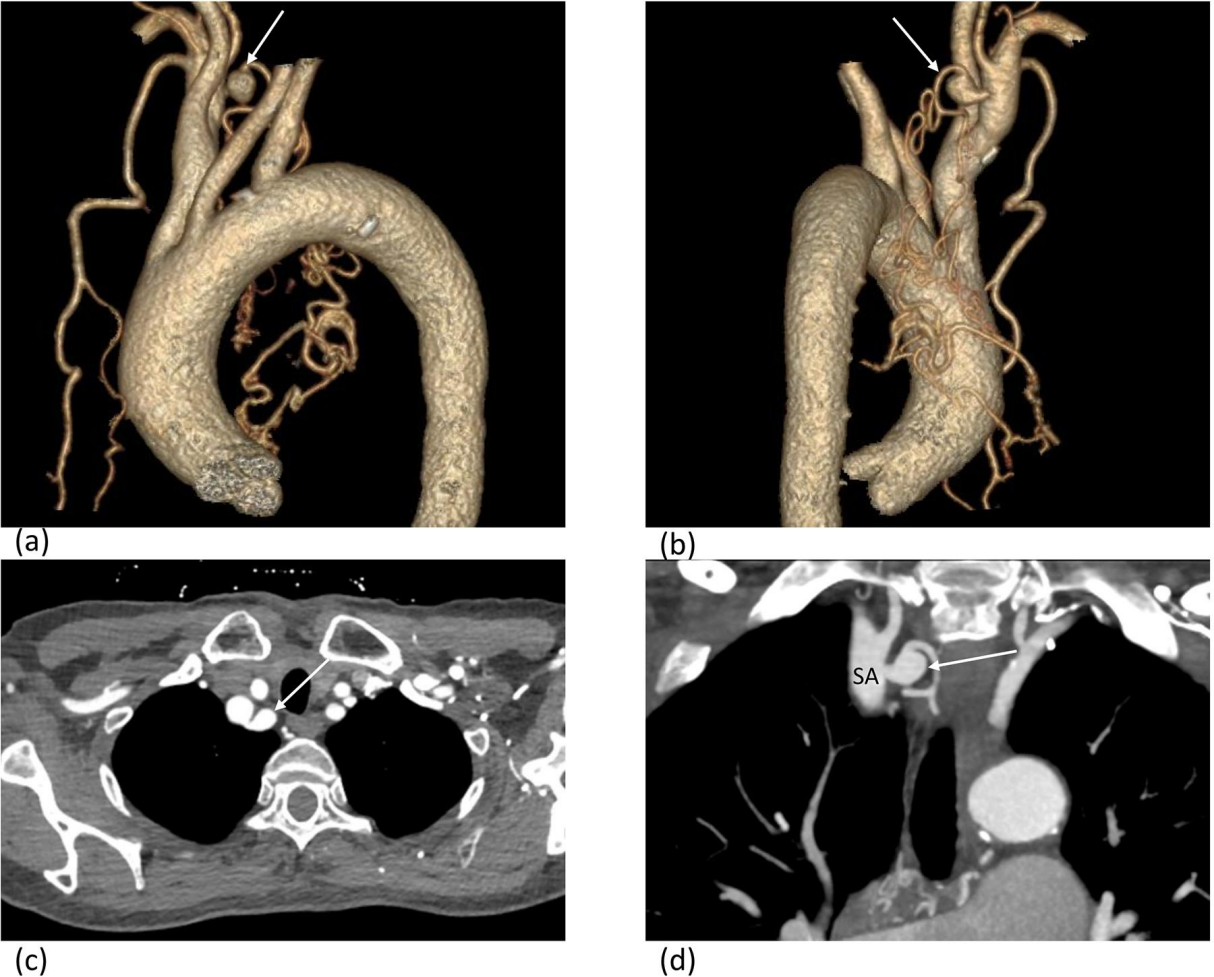
An aneurysm (white arrow) formation at the origin of the right subclavian artery branch vessels. 3D volume rendering **(a,b)**, axial **(c)** and coronal reconstruction **(d)** images show the aneurysm. SA subclavian artery.

CTPA imaging demonstrated interruption of the pulmonary artery in the right middle and lower lobes, with dilation of the pulmonary artery trunk and left pulmonary artery and filling defect in the left lower lobe pulmonary arteries (Figure 2). An aneurysm formation at the origin of the right subclavian artery branch vessels (Figure 3), with tortuosity and thickening of bilateral bronchial arteries and the right intercostal artery. Diagnosis: congenital interruption of the right middle and lower lobe pulmonary arteries with secondary collateral circulation formation and pulmonary hypertension; local small aneurysm formation at the origin of the right subclavian artery branch vessels. Widening of the pulmonary artery trunk and both pulmonary arteries. Distal pulmonary embolism on the left lower lobe pulmonary arteries.

### Therapeutic intervention and follow-up

The patient's vital signs were stable, and there was no hemoptysis. Consultation revealed no surgical indication for interruption of the pulmonary artery. Therapeutic interventions focused on controlling pulmonary hypertension and the conservative management of the right subclavian artery aneurysm. Assessment of pulmonary hypertension indicated the patient was in New York Heart Association Class II, with no decompensated right heart failure and no severe hypoxia. The right subclavian artery aneurysm was small in diameter, regular in shape, and free of dissection, mural thrombus, or risk of rapid enlargement or thrombus detachment. Given the patient's advanced age, stable condition, good compliance with medical advice, and strong preference for conservative management, anticoagulation therapy and antihypertensive medication were initiated, with regular follow-up scheduled.

During follow-up, the patient underwent regular outpatient evaluations every 6 months, including clinical assessment, blood pressure and lipid profile measurements, coagulation tests, echocardiography, neck CTA, electrocardiogram, and exercise tolerance testing. Clinical evaluation results indicated that the patient's blood pressure and lipid levels were stable, cardiac function was stable, and pulmonary arterial hypertension had not progressed. The neck CTA showed no enlargement or thrombus formation in the right subclavian artery aneurysm. The patient continued to adhere to medication therapy. No unexpected adverse events were observed during the follow-up period.

### Patient perspective

The patient was informed that she had a congenital cardiovascular abnormality and expressed concern and anxiety. She stated that she would follow her doctor's instructions by taking her medication on time, improving her lifestyle habits, and avoiding strenuous exercise and emotional ups and downs. During a follow-up visit six months later, she was told that her condition was stable and had not progressed. She expressed relief and said she was satisfied with her treatment. She experienced no adverse reactions during the diagnostic process and plans to continue with regular follow-up visits. Timeline of major clinical and nursing events were shown in Table 1.

**Table 1:** Timeline of major clinical and nursing events.

Date	Clinical Event/Status	Nursing & Medical Interventions
2025-02-27 (Admission)	The patient has had intermittent sleep disturbances and low mood for 20 years, with symptoms worsening over the past month. The patient experiences shortness of breath after physical activity and has a history of hypertension.	Admission Tests: Complete blood count, urinalysis, blood biochemical examination, coagulation function, chest CT scan, electrocardiogram, color Doppler ultrasound (abdominal, urinary tract, and cardiac).
2025-02-28 09:04	Elevated D-dimer levels. And the patient experiences shortness of breath after physical activity.	Perform a color Doppler ultrasound of the lower extremity veins, blood gas analysis, CT pulmonary angiography (CTPA), and Holter monitoring. Prescribe the following antidepressants and hypnotics: Trazodone hydrochloride tablets 50 mg orally once daily qn, Eszopiclone tablets 3 mg orally once daily qn, Olanzapine tablets 2.5 mg orally once daily qn, and Paroxetine hydrochloride enteric-coated sustained-release tablets 25 mg orally once daily qd.
2025-02-28 20:03	CTPA results suggest pulmonary embolism, interruption of pulmonary artery in the right middle and lower lobes and an aneurysm of the right subclavian artery.	Oxygen therapy, ECG monitoring; Anticoagulation and antihypertensive therapy. Anticoagulation: Rivaroxaban 15 mg po bid. Antihypertensive medications: Amlodipine besylate tablets 5 mg po qd, Irbesartan tablets 150 mg po qd.
2025-03-01	Re-evaluate coagulation function	Continue with the current treatment regimen
2025-03-03	D-dimer levels have returned to normal	Continue with the current treatment regimen
2025-03-04 (Discharge)	Symptoms have improved, the condition is stable and the patient is feeling well.	Discharge Plan; Discharge instructions for the patient: Oral anticoagulant rivaroxaban, oral antihypertensive medications amlodipine besylate tablets and irbesartan tablets, oral antidepressants and hypnotics. Follow-up appointment in 4 weeks for depression and sleep disorders, follow-up appointment in 6 months for pulmonary arterial hypertension and aneurysm.
2025-09-20	Follow-up	Outcome: The patient’s overall condition is acceptable. Ongoing care: Continue taking oral anticoagulants, antihypertensive medications, antidepressants and hypnotics. Follow-up every six months.

## Discussion

The embryological basis of pulmonary artery interruption is closely associated with developmental abnormalities of the sixth aortic arch. The intrapulmonary pulmonary arteries arise from the lung buds and the extrapulmonary pulmonary arteries arise from the proximal portion of the sixth aortic arch. It is thought to be caused by involution of the proximal sixth aortic arch, which destined to become the proximal pulmonary artery and persistence of the connection between the intrapulmonary artery and the distal sixth aortic arch, which eventually becomes the ductus arteriosus (3, 4, 6). In this case, the main trunk of the right pulmonary artery and the pulmonary artery of the right upper lobe are present, but the branches to the middle and lower lobes are absent. Consequently, the pulmonary arteries distal to the interruption site receive blood supply from residual systemic collateral circulation formed during the embryonic period. Whether there is a common embryological link between the interruption of the pulmonary artery and the aneurysm in the subclavian branch is worth exploring. The proximal part of the right subclavian artery is derived from the right fourth aortic arch. They are controlled by independent developmental programs of different pharyngeal arch segments. A localized developmental error in the sixth arch generally does not simultaneously ‘affect’ the fourth arch. However, there may be a hemodynamic influence, not a direct developmental chain reaction. Very early hemodynamic changes (such as the absence of one arch leading to compensatory dilation) may affect other arches (7). According to a systematic analysis by Wang et al. of 65 adult patients with Isolated unilateral absence of pulmonary artery (5), hemoptysis was the initial symptom in 41.5% of cases, recurrent respiratory infections were present in 35.4%, and exertional dyspnea was predominantly associated with pulmonary hypertension (PHT). Although DSA is considered the gold standard in imaging, CT angiography provides direct visualization of extensive systemic collateral circulation, clearly identifying the site of pulmonary artery interruption and the source of collateral vessels (e.g., bronchial arteries, intercostal arteries) (8). Chest CT can assess secondary changes in lung parenchyma (e.g., bronchiectasis, interstitial fibrosis). Key Points for Imaging Diagnosis CT Angiography (CTA): Direct Findings: Normal right pulmonary artery trunk; middle and lower lobe branches interrupted at the hilum with no distal filling. Indirect Findings: Reduced right lung volume with right mediastinal shift; enlarged collateral vessels (bronchial arteries, intercostal arteries) exhibiting a “comb-like” distribution (3, 5). Pulmonary parenchymal changes: bronchiectasis (30.2%), interstitial fibrosis (14.0%), or pulmonary bullae (14.0%) (5). Differential Diagnosis: Chronic Pulmonary Embolism: Thrombus organization with vascular stenosis, but the pulmonary artery trunk is often dilated, and D-dimer levels are elevated. Swyer-James Syndrome: Increased pulmonary transparency with small pulmonary arteries, but no collateral circulation. Polyarteritis nodosa: Vascular wall thickening with annular enhancement, predominantly involving the aorta and its branches. Clinical significance of collateral circulation: Following interruption of the right middle and lower lobe pulmonary arteries, compensatory proliferation of systemic collaterals (bronchial arteries, intercostal arteries, diaphragmatic arteries) occurs. However, these collateral vessels have thin walls prone to rupture, leading to hemoptysis (incidence 41.5%) (5). In this case, the patient did not present with hemoptysis and may be managed with long-term follow-up. Should relevant symptoms develop, multiple interventions or consideration of lobectomy may be required (6). Treatment strategies are summarized as follows: Conservative management is suitable for asymptomatic or mild PHT patients (9). Interventional embolization is effective for hemoptysis in the short term but has a high rate of collateral recanalization. Lobectomy is indicated for recurrent infections or massive hemoptysis but may lead to compensatory emphysema in the remaining lung. Vascular reconstruction is applicable when the distal pulmonary artery is well-developed, allowing for main pulmonary artery-distal pulmonary artery anastomosis, though it requires high technical proficiency (3, 5).

## Conclusion

Right middle-lower lobe pulmonary artery interruption represents a rare congenital vascular anomaly whose diagnosis relies on CT angiography. Collateral circulation formation is closely associated with hemoptysis and pulmonary hypertension, necessitating individualized treatment decisions that balance interventional, surgical, and pharmacological interventions. Early identification and long-term follow-up are critical for improving prognosis (2).

## Data Availability

The original contributions presented in the study are included in the article/Supplementary Material, further inquiries can be directed to the corresponding author.

## Supplementary material

SUPPLEMENTARY VIDEO 3D volume rendering reconstruction shows interruption of the pulmonary artery in the right middle and lower lobes (yellow) and pulmonary embolism on distal branch of the left lower lobe pulmonary arteries (red highlight).

## References

[1] Loo YJ Thomas R Yagnik L. Isolated unilateral absence of pulmonary artery (IUAPA) in adults: a case series and review of literature. Respirol Case Rep. (2023) 11(1):e01073. 10.1002/rcr2.1073 36523545 PMC9745554

[2] Taniguchi H Saito J Abo H Masaki Y Tsuda T Furuse H Isolated unilateral absence of the pulmonary artery. Am J Respir Crit Care Med. (2015) 192(4):518–9. 10.1164/rccm.201502-0209IM 26278797

[3] Kruzliak P Syamasundar RP Novak M Pechanova O Kovacova G. Unilateral absence of pulmonary artery: pathophysiology, symptoms, diagnosis and current treatment. Arch Cardiovasc Dis. (2013) 106(8-9):448–54. 10.1016/j.acvd.2013.05.004 23938302

[4] Williams EA Cox C Chung JH Grage RA Rojas CA. Proximal interruption of the pulmonary artery. J Thorac Imaging. (2019) 34(1):56–64. 10.1097/rti.0000000000000373 30376479

[5] Wang P Yuan L Shi J Xu Z. Isolated unilateral absence of pulmonary artery in adulthood: a clinical analysis of 65 cases from a case series and systematic review. J Thorac Dis. (2017) 9(12):4988–96. 10.21037/jtd.2017.11.49 29312703 PMC5756977

[6] Maini A Cousins S Holliday T Memoli JW Vasu TS Khaitan PG. Hemoptysis: unilateral pulmonary artery atresia? A case report. J Cardiothorac Surg. (2023) 18(1):199. 10.1186/s13019-023-02255-9 37386643 PMC10311879

[7] Mantri SS Raju B Jumah F Rallo MS Nagaraj A Khandelwal P Aortic arch anomalies, embryology and their relevance in neuro-interventional surgery and stroke: a review. Interv Neuroradiol. (2022) 28(4):489–98. 10.1177/15910199211039924 34516323 PMC9326868

[8] Bsharat RK Shehadeh D Atatrah RW Manasra MR Alhadad B Amr B. An isolated congenital absence of right pulmonary artery associated with coronary and multiple systemic collaterals: a case report and literature review. J Int Med Res. (2025) 53(2):3000605251320763. 10.1177/03000605251320763 39985208 PMC11846099

[9] Ten Harkel AD Blom NA Ottenkamp J. Isolated unilateral absence of a pulmonary artery: a case report and review of the literature. Chest. (2002) 122(4):1471–7. 10.1378/chest.122.4.1471 12377882

